# First-principles investigations of the controllable electronic properties and contact types of type II MoTe_2_/MoS_2_ van der Waals heterostructures

**DOI:** 10.1039/d4na00193a

**Published:** 2024-05-21

**Authors:** Son T. Nguyen, Nguyen V. Hieu, Huy Le-Quoc, Kien Nguyen-Ba, Chuong V. Nguyen, Huynh V. Phuc, Cuong Q. Nguyen

**Affiliations:** a Faculty of Electrical Engineering, Hanoi University of Industry Hanoi 100000 Vietnam nguyensontung@haui.edu.vn; b The University of Danang – University of Science and Education Da Nang 550000 Vietnam; c The University of Danang – University of Science and Technology Danang 550000 Vietnam nbkien@dut.udn.vn; d Department of Materials Science and Engineering, Le Quy Don Technical University Hanoi 100000 Vietnam; e Division of Theoretical Physics, Dong Thap University Cao Lanh 870000 Vietnam; f Institute of Research and Development, Duy Tan University Da Nang 550000 Vietnam nguyenquangcuong3@duytan.edu.vn; g Faculty of Natural Sciences, Duy Tan University Da Nang 550000 Vietnam

## Abstract

Two-dimensional (2D) van der Waals (vdW) heterostructures are considered as promising candidates for realizing multifunctional applications, including photodetectors, field effect transistors and solar cells. In this work, we performed first-principles calculations to design a 2D vdW MoTe_2_/MoS_2_ heterostructure and investigate its electronic properties, contact types and the impact of an electric field and in-plane biaxial strain. We find that the MoTe_2_/MoS_2_ heterostructure is predicted to be structurally, thermally and mechanically stable. It is obvious that the weak vdW interactions are mainly dominated at the interface of the MoTe_2_/MoS_2_ heterostructure and thus it can be synthesized in recent experiments by the transfer method or chemical vapor deposition. The construction of the vdW MoTe_2_/MoS_2_ heterostructure forms a staggered type II band alignment, effectively separating the electrons and holes at the interface and thereby extending the carrier lifetime. Interestingly, the electronic properties and contact types of the type II vdW MoTe_2_/MoS_2_ heterostructure can be tailored under the application of external conditions, including an electric field and in-plane biaxial strain. The semiconductor–semimetal–metal transition and type II–type I conversion can be achieved in the vdW MoTe_2_/MoS_2_ heterostructure. Our findings underscore the potential of the vdW MoTe_2_/MoS_2_ heterostructure for the design and fabrication of multifunctional applications, including electronics and optoelectronics.

## Abstract 2

Two-dimensional (2D) van der Waals (vdW) heterostructures are considered as promising candidates for realizing multifunctional applications, including photodetectors, field effect transistors and solar cells.

## Graphical abstract

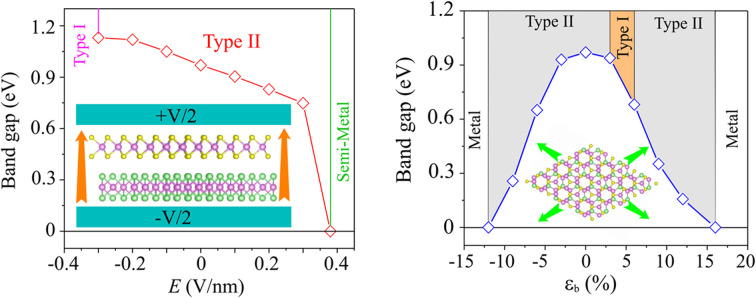

## 1 Introduction

The advent of two-dimensional (2D) materials, characterized by their atomic structures and unique properties, has ushered in a new era of exploration and innovation in condensed matter physics and materials science. This burgeoning class of materials, with graphene^1^ as a pioneering example, has captivated both fundamental and industrial researchers. Following the success of graphene, a plethora of 2D materials have been exfoliated and investigated, both in terms of fundamental understanding and practical applications. Recently, the most extensively investigated 2D materials are transition metal mono- and di-chalcogenides,^2–5^ MXenes^6–8^ and graphitic carbon nitrides.^9–11^ The versatility in the electronic and optical properties of 2D materials makes them promising candidates for multiple applications, ranging from advanced electronics to quantum computing and beyond.^12–15^

More interestingly, the special versatility of 2D materials lies in their potential for creating 2D vdW heterostructures by stacking them together.^16–18^ The 2D vdW heterostructures based on 2D materials offer an intriguing platform for tailoring and enhancing material properties, unlocking novel phenomena, and paving the way for practical applications in next-generation devices.^19–21^ Recently, a plethora of 2D vdW heterostructures have been synthesized experimentally and explored computationally, for instance, TMD heterostructures,^22,23^ MXene heterostructures,^24,25^ MA_2_Z_4_ heterostructures^26,27^ and phosphorene heterostructures.^28–30^ Among them, the exploration of vdW heterostructures between different 2D TMD materials has received much more consideration and interest. Many 2D TMD-based vdW heterostructures have been successfully fabricated and explored, such as MoS_2_/WSe_2_,^31,32^ HfS_2_/MoS_2_,^33^ MoS_2_/WS_2_,^34^ MoTe_2_/ReS_2_ (ref. 35) and black phosphorus/MoS_2_.^36^ One can find that the 2D vdW TMD heterostructures can be synthesized in experiments by various strategies, including top-down^37^ and bottom-up^38,39^ strategies.

Recently, a novel 2D vdW heterostructure based on MoTe_2_ and MoS_2_ TMD materials has been successfully fabricated in experiments by various methods, such as one-step CVD,^40^ mechanical exfoliation^41,42^ and direct imprinting.^43^ Using the one-step CVD technique, Ding *et al.*^40^ fabricated a MoTe_2_/MoS_2_ heterostructure and demonstrated that the photodetector based on such a heterostructure exhibits outstanding photoresponsivity and external quantum efficiency. Lately, Ji *et al.*,^41^ utilizing mechanical exfoliation, fabricated a 2D vdW MoTe_2_/MoS_2_ heterostructure. These findings proved that such a heterostructure can be considered as a promising candidate for optoelectronic devices and integrated photonics. In addition, the MoTe_2_/MoS_2_ heterostructure can also be fabricated by combining the mechanical exfoliation and transfer methods^42^ or direct imprinting.^43^ All these experimental findings highlighted the potential applications of MoTe_2_/MoS_2_ heterostructures for multifunctional devices, including electronics and optoelectronics. Despite experimental successes in the fabrication of MoTe_2_/MoS_2_ heterostructures, a comprehensive computational investigation into the depth of their atomic structure, electronic properties and the formation of contact types is notably lacking. Therefore, in this work, we perform first-principles calculations to design a MoTe_2_/MoS_2_ heterostructure and investigate its structures and electronic properties and the formation of type II band alignment. The impact of external conditions is also explored to examine the potential applications of the MoTe_2_/MoS_2_ heterostructure for multifunctional devices. Our findings underscore the potential of the vdW MoTe_2_/MoS_2_ heterostructure for the design and fabrication of multifunctional applications, including electronics and optoelectronics.

## 2 Computational methods

In this work, the first-principles calculations are performed using the Quantum Espresso simulation package.^44,45^ The geometric optimization process and electronic property calculations are performed in the framework of the Perdew–Burke–Ernzerhof (PBE) functional^46^ within the projector augmented-wave (PAW) pseudopotential.^47^ A cut-off energy of 510 eV and a Monkhorst–Pack (9 × 9 × 1) *K*-point mesh are employed for all the processes and calculations. The hybrid Heyd–Scuseria–Ernzerhof (HSE) functional^48^ is also employed to get a more accurate band gap value of materials. The weak vdW interactions that may occur in layered materials can be described by adding the long-range dispersion correction of the Grimme DFT-D3 method.^49^ The convergence threshold for the force and energy in all the calculations is set at 0.01 eV Å^−1^ and 10^−6^ eV, respectively. A vacuum thickness of 27 Å is applied along the *z* direction of materials to avoid any unnecessary interlayer interactions. A dipole correction is also employed for all the calculation processes.

## 3 Results and discussion

**Fig. 1:**
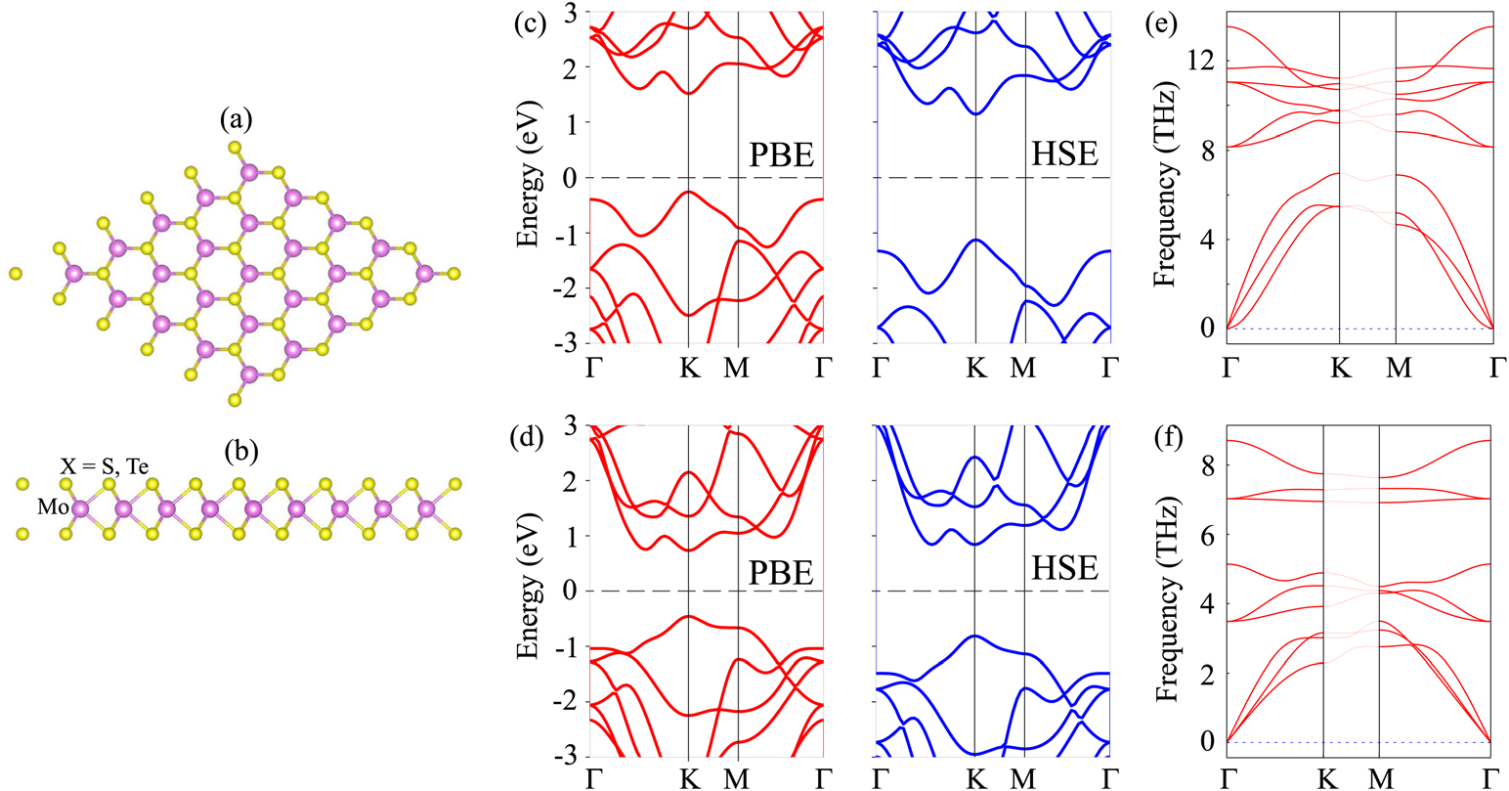
(a) Top view and (b) side view of the atomic structures of a MoX_2_ (X = S, Te) monolayer. Calculated electronic band structures of (c) MoS_2_ and (d) MoTe_2_ monolayers from PBE and HSE functionals. Phonon spectrum of (e) MoS_2_ and (f) MoTe_2_ monolayers.

We first investigate the atomic structure and electronic properties of MoX_2_ (X = Te, S) monolayers. The atomic structures of MoX_2_ are depicted in Fig. 1. The MoX_2_ monolayer consists of an X–Mo–X layer, where a Mo atom is sandwiched between two X atoms on different sides. Similar to graphene, the MoX_2_ monolayer shows a hexagonal atomic structure. The lattice parameters of MoTe_2_ and MoS_2_ monolayers are calculated to be 3.50 and 3.16 Å, respectively. These values are consistent with the experimental measurement.^50^ The electronic band structures of MoS_2_ and MoTe_2_ monolayers are depicted in Fig. 1(c) and (d). Both MoS_2_ and MoTe_2_ monolayers exhibit semiconducting behavior with a direct band gap. The valence band maximum (VBM) and conduction band minimum (CBM) are located at the *K* point for both the MoS_2_ and MoTe_2_ monolayers. The calculated band gaps of the MoS_2_ and MoTe_2_ monolayers are 1.78/2.26 and 1.19/1.65 eV given by the PBE/HSE functional, respectively. It is obvious that the traditional PBE functional underestimates the band gap of 2D materials, while the hybrid HSE06 can provide a more accurate band gap. However, both the PBE and HSE06 functionals yield consistent behavior for the MoS_2_ and MoTe_2_ monolayers. Hence, we employed the PBE functional for the subsequent calculations due to its low computational resource. The phononic spectrum of MoS_2_ and MoTe_2_ monolayers is illustrated in Fig. 1(e) and (f). One can observe that there are no negative frequencies in the phonon spectrum of both the MoS_2_ and MoTe_2_ monolayers, predicting that these monolayers are dynamically stable.

**Fig. 2:**
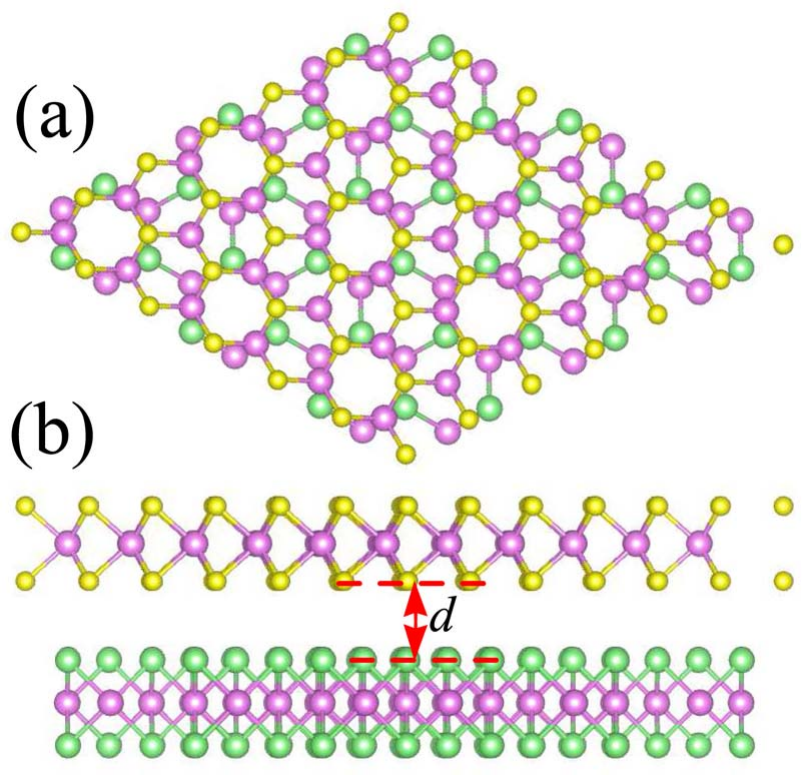
(a) Top view and (b) side view of the atomic structure of the MoTe_2_/MoS_2_ heterostructure.

The atomic structures of the MoTe_2_/MoS_2_ heterostructure are illustrated in Fig. 2. The MoTe_2_/MoS_2_ heterostructure is designed by using (2 × 2) unit cells of a MoS_2_ monolayer and 
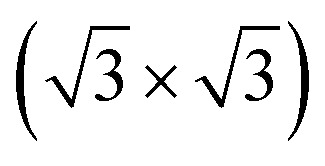
 unit cells of a MoTe_2_ monolayer. The lattice mismatch is calculated to be 2.1%, which is small and can be considered negligible. After geometric optimization, the interlayer spacing *d* between the two constituent MoTe_2_ and MoS_2_ monolayers is obtained to be 3.33 Å. This interlayer spacing is consistent with that in other typical vdW heterostructures^51–53^ and the experimental value.^42^ This indicates that the MoTe_2_/MoS_2_ heterostructure is characterized by weak vdW interactions. Furthermore, to examine the stability of the MoTe_2_/MoS_2_ heterostructure, we calculate the binding energy as follows:1
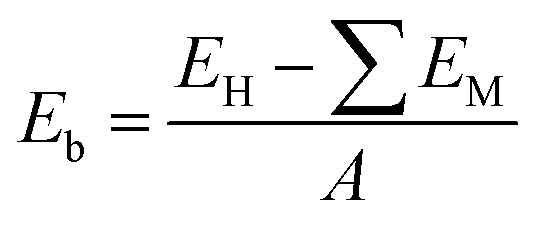
Here, *E*_H_ and *E*_M_ are the total energies of the MoTe_2_/MoS_2_ heterostructure and isolated MoX_2_ (X = S, Te) monolayers, respectively. *A* stands for the surface area of the heterostructure. The *E*_b_ of the MoTe_2_/MoS_2_ heterostructure is obtained to be −32.23 meV Å^−2^. The negative value of the binding energy indicates that the MoTe_2_/MoS_2_ heterostructure is structurally stable. In addition, we find that the value of the binding energy is consistent with that in graphite^54^ and other vdW-based systems.^55,56^ All these findings confirm that the weak vdW interactions are mainly dominated at the interface of the MoTe_2_/MoS_2_ heterostructure. It is noteworthy that the weak vdW interactions keep the MoTe_2_/MoS_2_ heterostructure stable and can be synthesized in recent experiments by the transfer method^42^ or chemical vapor deposition (CVD) method.^40^

**Fig. 3:**
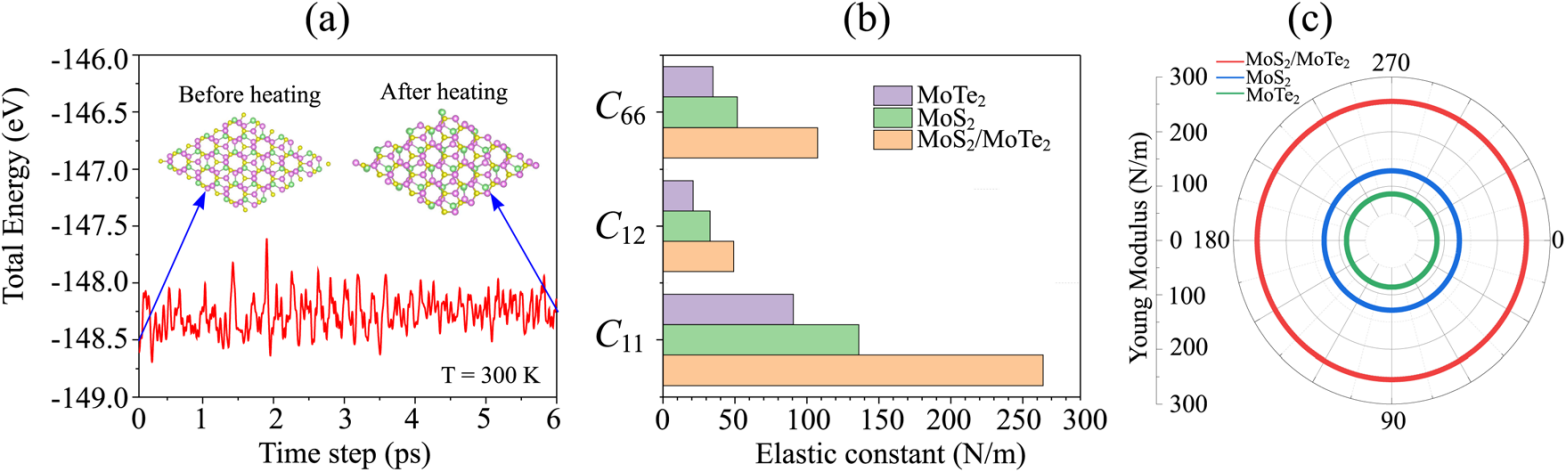
(a) The fluctuation in the total energy as a function of time steps of the MoTe_2_/MoS_2_ heterostructure. The insets show the atomic structures of the MoTe_2_/MoS_2_ heterostructure before and after heating for 6 ps. (b) Elastic constants and (c) angle-dependent Young's modulus of the MoTe_2_/MoS_2_ heterostructure and the isolated MoTe_2_ and MoS_2_ constituent monolayers.

Furthermore, to check the thermal and mechanical stability of the MoTe_2_/MoS_2_ heterostructure, we perform *Ab initio* molecular dynamics (AIMD) simulation and elastic constant calculation. The fluctuation in the total energy as a function of time steps of the MoTe_2_/MoS_2_ heterostructure is depicted in Fig. 3(a). It is evident that the change in the total energy of the MoTe_2_/MoS_2_ heterostructure before and after heating for 6 ps is small. Additionally, there is no distortion in the atomic structure of the MoTe_2_/MoS_2_ heterostructure after heating for 6 ps. All these findings confirm that the MoTe_2_/MoS_2_ heterostructure is thermally stable at room temperature of 300 K. The elastic constants *C*_*ij*_ of the MoTe_2_/MoS_2_ heterostructure are also calculated to evaluate its mechanical stability. The elastic constants of the MoTe_2_/MoS_2_ heterostructure are depicted in Fig. 3(b). The elastic constants of the constituent MoTe_2_ and MoS_2_ monolayers are also calculated for comparison. The calculated *C*_11_, *C*_12_ and *C*_66_ of the MoTe_2_/MoS_2_ heterostructure are 264.18, 49.17 and 107.50 N m^−1^, respectively. One can find that these values of the elastic constants satisfy the Born-Huang criteria,^57^ confirming that the heterostructure is mechanically stable. Furthermore, it is evident that the elastic constants of the MoTe_2_/MoS_2_ heterostructure exhibit a substantial enhancement compared to those in the constituent monolayers. Besides, the Young's modulus of the MoTe_2_/MoS_2_ heterostructure is also greater than that of the MoTe_2_ and MoS_2_ monolayers, exhibiting that the construction of the MoTe_2_/MoS_2_ heterostructure leads to an enhancement in the in-plane stiffness, as depicted in Fig. 3(c).

**Fig. 4:**
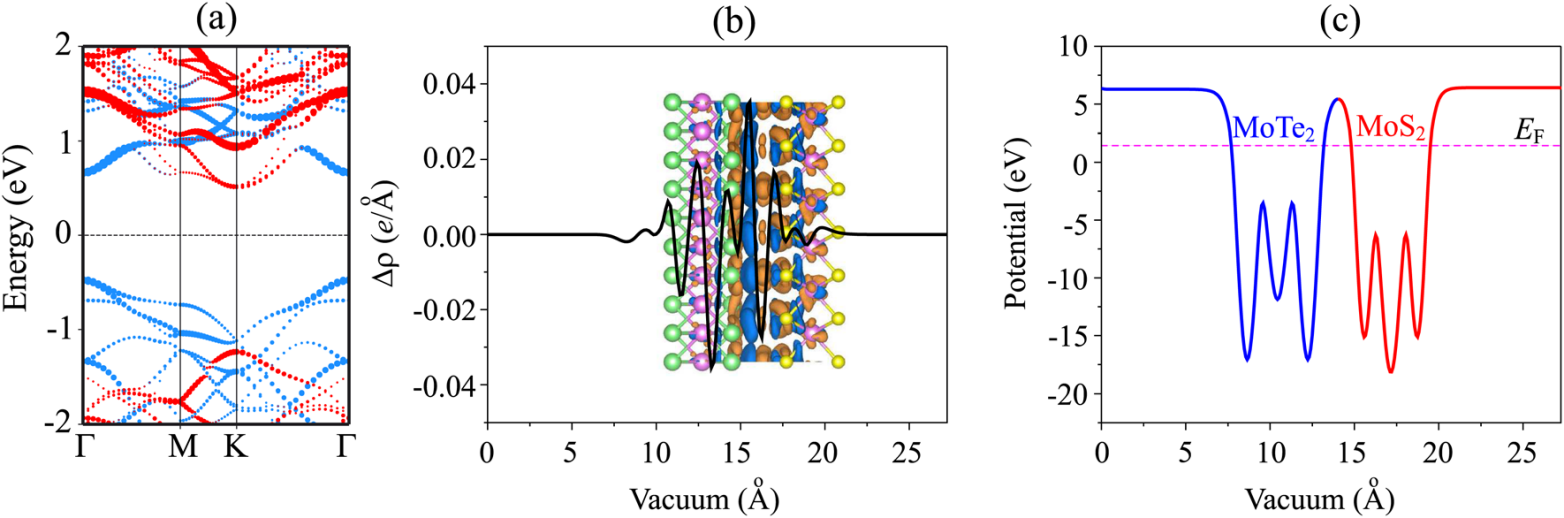
(a) Projected band structure, (b) planar-averaged charge density difference and (c) electrostatic potential of the MoTe_2_/MoS_2_ heterostructure. Red and blue balls stand for the contributions of the MoS_2_ and MoTe_2_ layers, respectively. The dark blue and dark orange represent the charge accumulation and depletion, respectively.

The projected band structure of the MoTe_2_/MoS_2_ heterostructure is depicted in Fig. 4(a). The MoTe_2_/MoS_2_ heterostructure possesses a semiconducting behavior with an indirect band gap. The VBM is located at the *Γ* point, whereas the CBM is at the *K* point. The band gap of the MoTe_2_/MoS_2_ heterostructure is 0.97 eV. Such a band gap is still smaller than that of both the constituent MoTe_2_ and MoS_2_ monolayers. This implies that the formation of the MoTe_2_/MoS_2_ heterostructure gives rise to a reduction in the band gap. A narrower band gap corresponds to stronger optical absorption. Therefore, the construction of the MoTe_2_/MoS_2_ heterostructure could lead to an enhancement in the optical properties. More interestingly, the band edges of the MoTe_2_/MoS_2_ heterostructure are contributed by distinct layers. The VBM is mainly contributed by the MoTe_2_ layer, while the CBM comes from the MoS_2_ layer. This finding suggests that the MoTe_2_/MoS_2_ heterostructure forms a type II band alignment. The formation of a type II band alignment was also observed in previous experiments.^41,42^ Therefore, the type II MoTe_2_/MoS_2_ heterostructure can be considered as a promising candidate for the design of optoelectronic and electronic devices, such as photodetectors and transistors.

We further consider the charge redistribution at the interface of the MoTe_2_/MoS_2_ heterostructure by analyzing the charge density difference (CDD) as follows:2
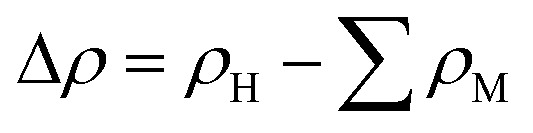
Here, *ρ*_H_ and *ρ*_M_ are the charge densities of the MoTe_2_/MoS_2_ heterostructure and isolated MoX_2_ (X = S, Se) monolayers, respectively. The planar-averaged CDD of the MoTe_2_/MoS_2_ heterostructure is depicted in Fig. 4(b). The dark blue and dark orange regions represent charge accumulation and depletion, respectively. It is evident that the charges are mainly accumulated in the MoTe_2_ layer and depleted in the MoS_2_ layer. It indicates that the MoTe_2_ gains electrons, while the MoS_2_ layer loses electrons. The electrons flow from the MoS_2_ to the MoTe_2_ layer, whereas the holes are transferred in the opposite direction, *i.e.* from the MoTe_2_ to the MoS_2_ layer. Bader charge analysis indicates that there is a small amount of charge transfer of about 10^−3^ electrons between the two constituent layers. Furthermore, to confirm the charge transfers in the MoTe_2_/MoS_2_ heterostructure, we also calculate the work functions of the MoTe_2_/MoS_2_ heterostructure and the constituent MoX_2_ monolayers. The work function of a material can be calculated as: *Φ* = *E*_vac_ − *E*_F_, where *E*_vac_ and *E*_F_ represent the vacuum energy and Fermi energy, respectively. The work functions of the MoTe_2_ and MoS_2_ monolayers are calculated to be 4.76 eV and 4.08 eV, respectively. The lower work function of the MoS_2_ monolayer compared to that of the MoTe_2_ monolayer confirms that the electrons move from the MoS_2_ to the MoTe_2_ layer upon the formation of the heterostructure. The work function of the MoTe_2_/MoS_2_ heterostructure is calculated to be 4.98 eV, which is larger than that of the MoS_2_ and MoTe_2_ layers. The electrostatic potential of the MoTe_2_/MoS_2_ heterostructure is displayed in Fig. 4(c). One can find that the difference in the potential of the MoS_2_ and MoTe_2_ layers is small, verifying a small amount of charge transfer between the two layers. In addition, the potential of the MoS_2_ layer is deeper than that of the MoTe_2_ layer, confirming that the electrons move from the MoS_2_ to the MoTe_2_ layer. Such charge transfer leads to the formation of a built-in electric field, pointing from the MoS_2_ to the MoTe_2_ layer in their combined heterostructure.

**Fig. 5:**
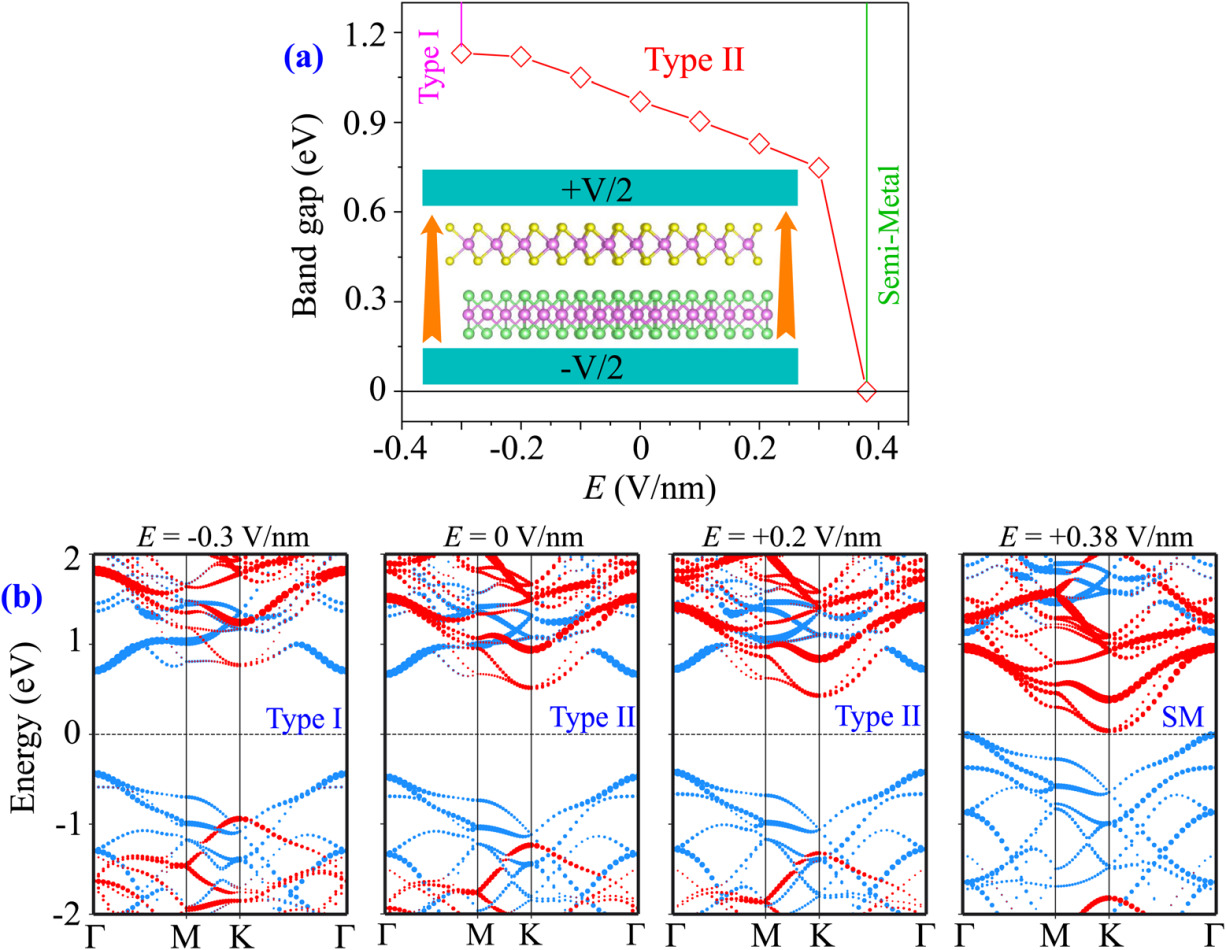
(a) The variation of the band gaps and (b) the projected band structures of the MoTe_2_/MoS_2_ heterostructure under electric fields of different strengths. The inset shows the schematic model of applied electric fields along the *z* direction of the heterostructure.

Furthermore, we examine how the external conditions impact the electronic properties and contact types of the MoTe_2_/MoS_2_ heterostructure. Therefore, external electric fields and biaxial strains are applied to the heterostructure. The electric fields are applied along the *z* direction of the heterostructure, as depicted in the inset of Fig. 5(a). The positive direction of the electric fields is defined as from the MoTe_2_ to the MoS_2_ layer in their combined heterostructure. It is evident that an electric field can be used to modify the band gaps and change the contact types in the MoTe_2_/MoS_2_ heterostructure, as shown in Fig. 5(a). The band gap of the MoTe_2_/MoS_2_ heterostructure increases with applying a negative electric field and decreases with applying a positive electric field. The physical mechanism of such change can be described as follows: the direction of the built-in electric field is opposite to that of the negative electric field. Thus, the negative electric field can give rise to an enhancement in the band gap of the MoTe_2_/MoS_2_ heterostructure because the total electric field is weakened. On the other hand, the direction of the built-in electric field and positive electric field is the same. The total electric field is strengthened. Thus, the positive electric field causes a reduction in the band gap of the MoTe_2_/MoS_2_ heterostructure. The negative electric field can also lead to the transition from type II to type I band alignment, while the positive electric field gives rise to the semiconductor to semimetal transition. The underlying mechanism of these transitions can be described by analyzing the projected band structures of the MoTe_2_/MoS_2_ heterostructure under electric fields of different strengths, as depicted in Fig. 5(b). At a critical strength of the negative electric field of −0.3 V nm^−1^, the CBM of the MoTe_2_/MoS_2_ heterostructure is located at the *Γ* point, as shown in Fig. 5(b). Such a CBM is contributed by the MoTe_2_ layer, indicating that the negative electric field gives rise to a shift in the CBM of the MoTe_2_/MoS_2_ heterostructure from the MoS_2_ to the MoTe_2_ layer. Meanwhile, the VBM of the MoTe_2_/MoS_2_ heterostructure remains at the *Γ* point and is contributed by the MoTe_2_ layer. These findings predict that the MoTe_2_/MoS_2_ heterostructure changes to form a type I band alignment. In addition, when a positive electric field is applied, both the CBM and VBM of the MoTe_2_/MoS_2_ heterostructure move towards the Fermi level, leading to a reduction in the band gap. At a critical strength of the positive electric field of +0.38 V nm^−1^, a transition from semiconductor to semimetal can be achieved in the MoTe_2_/MoS_2_ heterostructure, as its VBM crosses the Fermi level. Furthermore, it should be mentioned that a high strength electric field can be generated from the tabletop terahertz source within an electrolyte top gate.^58^ Additionally, a high strength electric field always requires high-*k* and a back (top)-gated device architecture.^59^ All these findings prove that the electric field can be considered as an effective tool to manipulate the electronic properties and contact type of the MoTe_2_/MoS_2_ heterostructure, thereby expanding its potential applications in electronics and optoelectronics.^60^

**Fig. 6:**
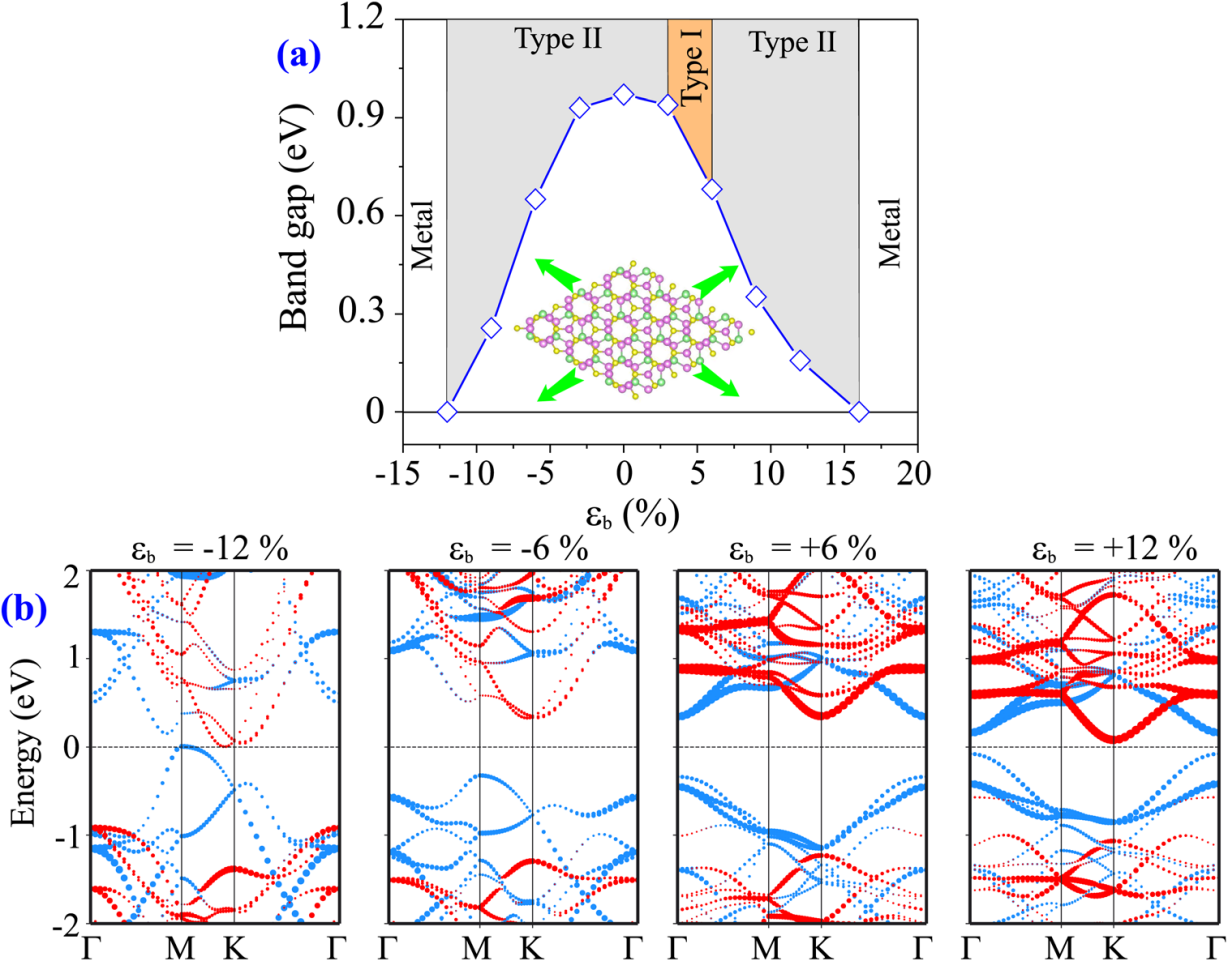
(a) The variation of the band gaps and (b) the projected band structures of the MoTe_2_/MoS_2_ heterostructure under different ratios of strain. The inset shows the schematic model of the applied in-plane biaxial strain to the heterostructure.

The biaxial in-plane strain is derived from *ε*_b_ = (*a* − *a*_0_)/*a*_0_ × 100%, where *a* and *a*_0_ are the lattice parameters of the MoTe_2_/MoS_2_ heterostructure with and without the application of the biaxial strain, respectively. The negative and positive values refer to the compressive and tensile strains, respectively. The schematic model of the in-plane biaxial strain is depicted in the inset of Fig. 6. It is obvious that the strain causes a change in both the band gap values and contact types of the MoTe_2_/MoS_2_ heterostructure. The biaxial strain gives rise to a reduction in the band gap of the MoTe_2_/MoS_2_ heterostructure, as depicted in Fig. 6(a). The band gap of the MoTe_2_/MoS_2_ heterostructure can be reduced down to zero under the application of either a compressive strain of −12% or a tensile strain of +16%. This observation indicates that the transition from semiconductor to metal can be achieved in the MoTe_2_/MoS_2_ heterostructure under the application of biaxial strain. Additionally, the tensile strain can also lead to the transformation between type II and type I band alignment in the MoTe_2_/MoS_2_ heterostructure.

To have a better understanding of the impact of the strain, we further analyze the projected band structures of the MoTe_2_/MoS_2_ heterostructure under different strain ratios, as illustrated in Fig. 6(b). When the compressive strain is applied, both the VBM and CBM of the MoTe_2_/MoS_2_ heterostructure shift towards the Fermi level, giving rise to a reduction in the band gap values. Similarly, the band edges of both the MoTe_2_ and MoS_2_ layers in the MoTe_2_/MoS_2_ heterostructure move closer to the Fermi level under the tensile strain. Under a tensile strain of *ε*_b_ = +3%, the CBM of the MoTe_2_/MoS_2_ heterostructure shifts from the *K* to the *Γ* point. Thus, the indirect-to-direct transition is achieved in the MoTe_2_/MoS_2_ heterostructure. In addition, both the VBM and CBM of the MoTe_2_/MoS_2_ heterostructure now come from the MoTe_2_ layer, indicating that there occurs a transition from type II to type I band alignment. The type I band alignment is maintained in the MoTe_2_/MoS_2_ heterostructure under tensile strains ranging from +3% to +6%. When the tensile strain is larger than +6%, the CBM of the MoTe_2_/MoS_2_ heterostructure is recovered from the *Γ* to the *K* point, while the VBM is preserved at the *Γ* point. This recovery indicates that there is a transition from direct to indirect semiconductor and a conversion from type I to type II band alignment because the band edges of the MoTe_2_/MoS_2_ heterostructure are contributed by the MoTe_2_ layer. When the tensile strain is larger than 16%, the band edges of the MoTe_2_/MoS_2_ heterostructure cross the Fermi level, leading to a transition from semiconductor to metal. Our findings prove that the in-plane biaxial strain can effectively be used to tailor the electronic properties and contact types in the MoTe_2_/MoS_2_ heterostructure, thereby expanding its potential applications in multifunctional devices.

## 4 Conclusions

In conclusion, we have performed first-principles calculations to design a 2D vdW MoTe_2_/MoS_2_ heterostructure with the formation of a type II band alignment. Our results indicate that the MoTe_2_/MoS_2_ heterostructure is structurally, thermally and mechanically stable. The weak vdW interactions are found to be dominated at the interface of the MoTe_2_/MoS_2_ heterostructure and thus it can be synthesized in recent experiments by the transfer method or chemical vapor deposition. The vdW MoTe_2_/MoS_2_ heterostructure exhibits a staggered type II band alignment, effectively separating the electrons and holes at the interface and thereby extending the carrier lifetime. Furthermore, our findings reveal that the electronic properties and contact types of type II vdW MoTe_2_/MoS_2_ heterostructures can be tailored under the application of external conditions, including an electric field and in-plane biaxial strain. The semiconductor–semimetal–metal transition and type II–type I conversion can be achieved in the vdW MoTe_2_/MoS_2_ heterostructure. Our findings underscore the potential of the vdW MoTe_2_/MoS_2_ heterostructure for the design and fabrication of multifunctional applications, including electronics and optoelectronics.

## Conflicts of interest

There are no conflicts to declare.
